# Supplementary material for: Scaling mental health care in Nigeria: Impact of WHO mhGAP training under the MeHPriC program on knowledge, attitudes, and practices of primary health care workers in Lagos State – A pre-post mixed-methods study
Source: Glob Ment Health (Camb). 2025 Jul 17;12:e83. doi: 10.1017/gmh.2025.10040 (PMC12322782; doi:10.1017/gmh.2025.10040)
Supplement: Adewuya et al. supplementary material [file S205442512510040Xsup001.docx]

**Supplementary File S1: Adapted Instruments**

**1. mhGAP Knowledge Questionnaire**

- **Description**: A 25-item multiple-choice tool, adapted from the WHO mhGAP Monitoring and Evaluation Toolkit (WHO, 2018), assessing knowledge of five priority conditions: depression, other significant mental health complaints (including anxiety-related symptoms), psychosis, epilepsy, and suicide risk. Fifteen items evaluate factual knowledge (e.g., symptoms, risk factors, diagnosis), and ten are clinical vignettes requiring algorithm-based decision-making, aligned with the mhGAP Intervention Guide (WHO, 2016).
- **Scoring**: Correct responses score 1 point, incorrect 0 points (range: 0–25). Categories: Low (0–10), Moderate (11–20), High (≥21).
- **Reliability**: Cronbach’s α = 0.82 (pilot-tested with 30 PHC workers, April 2017).
- **Sample Item** (Item 1): “What are the core symptoms of moderate depression according to the mhGAP-IG?”

Options:

- - A. Persistent sadness, loss of interest, fatigue;
  - B. Hallucinations, delusions;
  - C. Seizures, loss of consciousness;
  - D. Excessive worry, panic attacks (Correct: A).
- **Items** (25 total, 5 per condition):

1. What are the core symptoms of moderate depression according to the mhGAP-IG? (A–D
2. Which medication is first-line for epilepsy in PHC settings? (A–D)
3. What is the initial step in assessing suicide risk per mhGAP guidelines? (A–D
4. How should psychosis be differentiated from spiritual possession in PHC? (A–D)
5. What psychoeducation is appropriate for other significant mental health complaints (e.g., anxiety-related symptoms)? (A–D)
6. Which symptoms indicate a need for urgent referral in psychosis cases? (A–D)
7. What is the recommended follow-up schedule for depression management? (A–D)
8. Which antiepileptic drug is contraindicated in pregnancy? (A–D)
9. How should a PHC worker respond to a patient expressing suicidal ideation? (A–D)
10. What are common triggers for anxiety-related complaints in Nigeria? (A–D)

11–15. [Factual items on epidemiology, risk factors, and management]

16–20. [Vignettes on depression, psychosis, epilepsy]

21–25. [Vignettes on suicide risk, anxiety-related complaints]

- **Notes**: Pilot-testing showed 90% correct response rate for epilepsy items, reflecting high local prevalence, compared to 70% for suicide risk, indicating baseline gaps (Section 3.2).

**2. Stigma and Attitude Scale**

- **Description**: A 15-item Likert-scale, adapted from the WHO mhGAP Monitoring and Evaluation Toolkit (WHO, 2018) and the Mental Illness: Clinicians’ Attitudes (MICA) Scale (Gabbidon et al., 2013), measuring beliefs about the treatability, danger, social acceptability, and moral attributions of mental, neurological, and substance use (MNS) disorders.
- **Scoring**: 1=Strongly Disagree, 5=Strongly Agree (range: 15–75). Higher scores indicate greater stigma. Categories: Low (15–30), Moderate (31–54), High (≥55). Reverse-scored items: 3, 7, 12.
- **Reliability**: Cronbach’s α = 0.78 (pilot-tested with 30 PHC workers).
- **Sample Item** (Item 1): “People with psychosis are dangerous and should be isolated.” (1=Strongly Disagree, 5=Strongly Agree).
- **Items** (15 total, 3 per condition):
  1. People with psychosis are dangerous and should be isolated.
  2. Epilepsy is caused by non-medical (e.g., spiritual) forces.
  3. Depression can be treated effectively in PHC settings (reverse-scored).
  4. Suicide risk is a sign of personal weakness.
  5. Other significant mental health complaints (e.g., anxiety-related symptoms) are not real medical issues.
  6. Patients with psychosis cannot integrate into community life.
  7. Epileptic patients can lead normal lives with treatment (reverse-scored).
  8. Depression is a choice and reflects lack of willpower.
  9. Suicidal patients are attention-seeking.
  10. Anxiety-related complaints are exaggerated and untreatable.
  11. Psychosis requires hospitalization rather than PHC care.
  12. PHC workers can manage epilepsy effectively (reverse-scored).
  13. Depressed patients burden the PHC system.
  14. Suicide risk cannot be managed in PHC settings.
  15. Anxiety-related complaints are due to spiritual imbalance.
- **Notes**: High baseline stigma for psychosis (mean=4.2) reduced post-training (d=0.52, Table 2), but persistent cultural beliefs were noted (Supplementary Table S1).

**3. Self-Reported Practice Change Survey**

- **Description**: A 10-item Likert-scale, adapted from the WHO mhGAP Monitoring and Evaluation Toolkit (WHO, 2018) and Kirkpatrick’s Level 3 evaluation (Kirkpatrick & Kirkpatrick, 2006), assessing the frequency of mhGAP-aligned behaviors at 5-month follow-up, including screening, diagnosis, psychoeducation, and referral.
- **Scoring**: 1=Never, 5=Always (range: 10–50). Categories: Low (10–24), Moderate (25–34), Enhanced (≥35). The ≥35 threshold reflects consistent engagement (average item score ≥3.5, indicating “Often” or “Always”), established through pilot-testing with 30 PHC workers and validated by expert consultation with Nigerian mental health specialists (WHO, 2018).
- **Reliability**: Cronbach’s α = 0.85.
- **Sample Item** (Item 1): “During routine patient consultations, I actively screen for mental health problems by asking about mood, anxiety, or emotional difficulties.” (1=Never, 5=Always).
- **Items** (10 total, 2 per condition):
  1. During routine patient consultations, I actively screen for mental health problems by asking about mood, anxiety, or emotional difficulties.
  2. I use mhGAP flowcharts to diagnose depression in patients presenting with low mood.
  3. I refer patients with complex psychosis to psychiatric specialists when indicated.
  4. I provide psychoeducation to epilepsy patients about medication adherence.
  5. I assess suicide risk by asking direct questions about suicidal thoughts or plans.
  6. I document mental health cases using mhGAP forms in patient records.
  7. I counsel patients with other significant mental health complaints (e.g., anxiety-related symptoms) using mhGAP guidelines.
  8. I monitor depression patients’ progress during follow-up visits.
  9. I collaborate with colleagues to manage epilepsy cases in PHC settings.
  10. I refer suicidal patients to appropriate support services when needed.
- **Notes**: 69.1% achieved enhanced practice (Table 2), with high screening rates (82.3%) linked to flowchart use (Section 3.4).

**4. Self-Efficacy Scale**

- **Description**: A 10-item Likert-scale, adapted from the WHO mhGAP Monitoring and Evaluation Toolkit (WHO, 2018) and Bandura’s self-efficacy framework (Bandura, 2006), assessing confidence in applying mhGAP protocols at 5-month follow-up, including diagnosis, management, and referral.
- **Scoring**: 1=Not at all confident, 5=Extremely confident (range: 10–50). Categories: Low (10–24), Moderate (25–34), High (35–44), Very High (45–50).
- **Reliability**: Cronbach’s α = 0.80.
- **Sample Item** (Item 1): “How confident are you in using the mhGAP flowchart to diagnose depression in a patient presenting with low mood?” (1=Not at all confident, 5=Extremely confident).
- **Items** (10 total, 2 per condition):
  1. I am confident using the mhGAP flowchart to diagnose depression in a patient presenting with low mood.
  2. I am confident managing epilepsy cases in PHC settings using mhGAP protocols.
  3. I am confident assessing suicide risk by asking direct questions about suicidal thoughts.
  4. I am confident differentiating psychosis from other conditions in PHC settings.
  5. I am confident counseling patients with other significant mental health complaints (e.g., anxiety-related symptoms).
  6. I am confident referring complex depression cases to specialists when needed.
  7. I am confident monitoring epilepsy patients’ medication adherence.
  8. I am confident responding to patients expressing suicidal ideation.
  9. I am confident managing psychosis cases with mhGAP flowcharts.
  10. I am confident providing psychoeducation for anxiety-related complaints.
- **Notes**: High self-efficacy (mean=42.15, Section 3.5) was reported for depression (88.4%) and epilepsy (85.7%), lower for anxiety-related complaints (70.3%), reflecting training focus.

**Supplementary File S2: Qualitative Interview and FGD Guides**

**FGD Guide (English)**

- **Introduction**: Welcome participants, explain the study purpose (evaluate mhGAP training impact), ensure confidentiality, and note the 60–90-minute duration. Obtain written consent for audio-recording, as per ethical approval (Section 2.7).
- **Prompts (CFIR Domains)**:
  - **Individual Characteristics**:
    1. How has the mhGAP training changed your confidence in managing mental health conditions like depression, psychosis, or epilepsy? (Probe: diagnostic skills, patient communication, use of flowcharts)
    2. Have your attitudes toward patients with psychosis, epilepsy, or suicide risk changed since the training? (Probe: empathy, stigma, interactions with patients/families)
  - **Inner Setting**:
    1. What challenges do you face in applying mhGAP protocols in your PHC facility? (Probe: medication availability, consultation space, workload, documentation)
    2. How do PHC resources (e.g., staff, tools, referral pathways) support or hinder mhGAP implementation? (Probe: availability of psychotropic drugs, specialist access)
  - **Process**:
    1. How have monthly supervision visits and WhatsApp peer support groups supported your mhGAP implementation? (Probe: case reviews, troubleshooting, frequency of engagement)
    2. What role do job aids (e.g., flowcharts, referral forms) play in your daily practice? (Probe: usability, accessibility)
  - **Outer Setting**:
    1. What additional training or resources would help scale mhGAP services in Lagos State? (Probe: child mental health, substance use, mentorship roles)
    2. How do community perceptions of mental health affect your ability to implement mhGAP? (Probe: patient uptake, family support)
- **Closing**: Summarize key points, invite additional comments, and thank participants for their contributions.

**KII Guide (English)**

- **Introduction**: Welcome interviewee, explain the study purpose (evaluate mhGAP training and implementation), ensure confidentiality, and note the 45–60-minute duration. Obtain written consent for audio-recording, as per ethical approval (Section 2.7).
- **Prompts (CFIR Domains)**:
  - **Intervention Characteristics**:
    1. How feasible was the mhGAP training delivery for PHC workers in Lagos State? (Probe: duration, content relevance, training methods)
    2. What aspects of the training curriculum were most effective or challenging? (Probe: role-plays, flowcharts, cultural adaptations)
  - **Inner Setting**:
    1. How well is mhGAP integrated into existing PHC systems in Lagos State? (Probe: coordination with LCDA coordinators, resource allocation, documentation)
    2. What PHC system factors support or hinder mhGAP implementation? (Probe: staffing, psychotropic drug supply, referral pathways)
  - **Process**:
    1. How effective were monthly supervision visits and WhatsApp peer support groups in supporting mhGAP implementation? (Probe: frequency, case review quality, digital engagement)
    2. What monitoring mechanisms ensure mhGAP protocol fidelity in PHCs? (Probe: supervision checklists, reporting systems)
  - **Outer Setting**:
    1. What policy or resource changes would enhance mhGAP scalability in Lagos State? (Probe: funding, specialist access, community engagement)
    2. How do state-level policies or community attitudes influence mhGAP implementation? (Probe: Lagos State Mental Health Policy, stigma)
- **Closing**: Summarize key points, invite additional comments, and thank the interviewee for their insights.

**Supplementary Table S1: Additional Qualitative Quotes and CFIR Alignments**

| **CFIR Domain** | **Theme** | **Sub-Theme** | **Quote** | **Source** | **Quantitative Convergence** |
| --- | --- | --- | --- | --- | --- |
| Individual Characteristics | Increased Clinical Confidence | Diagnostic Skills | “Before, I was scared to ask about suicide. Now, I can ask directly and know what to do.” | Nurse, Rural PHC | Knowledge gain: suicide risk d=0.85 (Table 2) |
| Individual Characteristics | Increased Clinical Confidence | Communication Skills | “I use the flowchart to talk to patients calmly, even with psychosis. It makes them trust me.” | Doctor, Urban PHC | Self-efficacy: mean=42.15, psychosis 78.5% (Section 3.5) |
| Individual Characteristics | Stigma Reduction and Empathy | Medical Etiology Understanding | “We used to avoid epileptics. Now we understand it’s a medical condition, not spiritual.” | Community Health Officer, Urban PHC | Stigma reduction: epilepsy d=0.49 (Table 2) |
| Individual Characteristics | Stigma Reduction and Empathy | Persistent Cultural Beliefs | “Some still think psychosis is spiritual, but we now counsel families to seek PHC care.” | Social Worker, Rural PHC | Stigma: 19.5% high stigma at follow-up (Table 2) |
| Individual Characteristics | Stigma Reduction and Empathy | Empathy Building | “I feel for depressed patients now; they’re not just lazy, they need help.” | Nurse, Peri-Urban PHC | Stigma: depression d=0.35 (Table 2) |
| Inner Setting | System-Level Challenges | Medication Stock-Outs | “Flowcharts help, but we lack drugs like antipsychotics in our PHC.” | Doctor, Peri-Urban PHC | Practice: 69.1% enhanced, referral 74.8% (Table 2) |
| Inner Setting | System-Level Challenges | Workload Pressures | “We’re busy with deliveries; mental health screening takes extra time.” | Nurse, Urban PHC | Practice variability: non-clinical 55.8% enhanced (Section 3.4) |
| Inner Setting | System-Level Challenges | Space Constraints | “No private room for counseling; patients feel shy to talk about anxiety.” | Counselor, Rural PHC | Practice: psychoeducation 71.2% (Section 3.4) |
| Process | Role of Supervision and Peer Support | Case Review Effectiveness | “Supervisors reviewed cases monthly, helping us manage psychosis better.” | Midwife, PHC | Supervision OR=4.3 (Table 3) |
| Process | Role of Supervision and Peer Support | WhatsApp Troubleshooting | “I shared a psychosis case on WhatsApp; trainers advised quickly on referral.” | Community Health Officer, Rural PHC | WhatsApp OR=2.1 (Table 3) |
| Outer Setting | Desire for Expansion and Mentorship | Child Mental Health Modules | “We want mhGAP for children and substance use, to help more patients.” | Community Health Officer, Rural PHC | Practice: 69.1% enhanced (Table 2) |
| Outer Setting | Desire for Expansion and Mentorship | Mentorship Readiness | “I can train others on epilepsy now, if given support and resources.” | Doctor, Urban PHC | Self-efficacy: epilepsy 85.7% (Section 3.5) |

**Notes**:

- Quotes are derived from 12 FGDs (n=84, 6–8 participants each, balanced by cadre and urban/rural LCDAs) and 10 KIIs (5 LCDA coordinators, 3 master trainers, 2 Lagos State Ministry of Health officials),
- Thematic analysis was guided by CFIR , with coding by two independent analysts achieving high inter-rater reliability (kappa=0.82).

**Sample Yoruba translation of the Instruments**

**1. mhGAP Knowledge Questionnaire (Ìbéèrè ìmọ̀ nípa mhGAP)**

| **English Item** | **Yoruba Translation** |
| --- | --- |
| What are the core symptoms of moderate depression according to the mhGAP-IG? | Kí ni àwọn ààmì pàtàkì tí ìbànújẹ àárín gbùngbùn ní, gẹ́gẹ́ bí mhGAP-IG ṣe sọ? |
| Which medication is first-line for epilepsy in PHC settings? | Ẹ̀sùn wo ni wọ́n gbọ́dọ̀ fi bẹ̀rẹ̀ itọju àìlera ọpọlọ (epilepsy) ní ilé ìwòsàn PHC? |
| What is the initial step in assessing suicide risk per mhGAP guidelines? | Kí ni ìgbésẹ̀ àkọ́kọ́ láti fojú inú wo ewu ìpá-ẹni-ní-kú gẹ́gẹ́ bí mhGAP ṣe sọ? |
| How should psychosis be differentiated from spiritual possession in PHC? | Báwo ni a ṣe lè yà ìbàjẹ́ ọpọlọ (psychosis) kúrò lọ́dọ̀ àgbọ̀wọ̀ ẹ̀mí (possessions)? |
| What psychoeducation is appropriate for anxiety-related complaints? | Ìmọ̀ ẹ̀kọ́ wo ni ó yẹ fún àǹfààní àwọn oní ìbànújẹ tàbí ìfọ̀kànbalẹ̀? |
| Which symptoms indicate a need for urgent referral in psychosis cases? | Àmì wo ni fihan pé a nílò fífi aláìlera ọpọlọ ránṣẹ́ síbi tó yẹ lẹsẹkẹsẹ́? |
| What is the recommended follow-up schedule for depression management? | Kí ni àkókò tí a gbọ́dọ̀ máa tẹ̀síwájú ṣàbẹ̀wò fún àtọ́kànwá ìbànújẹ? |
| Which antiepileptic drug is contraindicated in pregnancy? | Èwo nínú àwọn olóògùn epilepsy ni a kò gbọdọ̀ fi fún aboyún? |
| How should a PHC worker respond to a patient expressing suicidal thoughts? | Báwo ni oṣiṣẹ́ PHC ṣe yẹ kí ó dá ẹni tí ń sọ̀rọ̀ ìpá-ẹni-ní-kú lóhùn? |
| What are common triggers for anxiety-related complaints in Nigeria? | Kí ni àwọn nǹkan tí wọ́n sábà máa fa ìfọ̀kànbalẹ̀ tó pọ̀ jù lọ ní Nàìjíríà? |

**2. Stigma and Attitude Scale (Ìwọn ìdálẹ́kùn àti ìfèsọ́nà)**

| **English Item** | **Yoruba Translation** |
| --- | --- |
| People with psychosis are dangerous and should be isolated. | Àwọn ẹni tí wọn ní psychosis lewu, a sì gbọ́dọ̀ yà wọn sọ́tọ̀. |
| Epilepsy is caused by non-medical (e.g., spiritual) forces. | Ẹ̀mí àìlera ọpọlọ (epilepsy) jẹ́ iṣẹ́ agbára aláìlera tàbí ẹ̀mí. |
| Depression can be treated effectively in PHC settings. *(Reverse)* | A lè ṣe itọju ìbànújẹ dáadáa ní ilé ìwòsàn PHC. |
| Suicide risk is a sign of personal weakness. | Ẹ̀mí ìpá-ẹni-ní-kú fihan pe ẹni naa rọrùn jẹ. |
| Anxiety-related complaints are not real medical issues. | Ìfọ̀kànbalẹ̀ kò jẹ́ àìlera gidi. |
| Patients with psychosis cannot integrate into community life. | Àwọn aláìlera psychosis kò le bá ìjọba tàbí àdúgbò ṣiṣẹ́ pọ̀. |
| Epileptic patients can lead normal lives with treatment. *(Reverse)* | Àwọn ẹni tí wọn ní epilepsy lè gbé ayé àìlera bí wọ́n bá ní itọju to peye. |
| Depression is a choice and reflects lack of willpower. | Ìbànújẹ jẹ́ yíyan ara ẹni, tó sì fi hàn pé ẹni naa kò ní agbára ọkàn. |
| Suicidal patients are attention-seeking. | Àwọn ẹni tí ń ronú ìpá-ẹni-ní-kú ń fẹ́ kí a máa tọ́jú wọn lẹ́kúnrẹ́rẹ́. |
| Anxiety-related complaints are exaggerated and untreatable. | Àwọn ìfọ̀kànbalẹ̀ jẹ́ àtẹ̀sà àti pé a kì í lè tọju wọn. |
| Psychosis requires hospitalization rather than PHC care. | A gbọ́dọ̀ gba àwọn oní psychosis wọ̀lé sí ilé ìwòsàn tó gaju ju PHC lọ. |
| PHC workers can manage epilepsy effectively. *(Reverse)* | Àwọn oṣiṣẹ́ PHC lè ṣe ìtọju epilepsy dáadáa. |
| Depressed patients burden the PHC system. | Àwọn oní ìbànújẹ ń dẹrù ba eto ilé ìwòsàn PHC. |
| Suicide risk cannot be managed in PHC settings. | Ẹ̀mí ìpá-ẹni-ní-kú kì í ṣe ohun tí PHC le tọju. |
| Anxiety-related complaints are due to spiritual imbalance. | Ìfọ̀kànbalẹ̀ jẹ́ nítorí àìdọ̀gba ẹ̀mí. |

**3. Self-Reported Practice Change Survey (Ìwádìí Ayípadà Ìṣe Tí Olùkànsí Ràpadà Lẹ́nu)**

| **English Item** | **Yoruba Translation** |
| --- | --- |
| I actively screen for mental health problems. | Mo máa ń ṣàyẹ̀wò àwọn àìlera ọpọlọ lójoojúmọ́. |
| I use mhGAP flowcharts to diagnose depression. | Mo máa ń lò àtòsọ́nà mhGAP láti mọ ìbànújẹ. |
| I refer psychosis cases to psychiatric specialists. | Mo máa ń rán àwọn oní psychosis lọ sí agbẹ́jọ́rò ọpọlọ. |
| I give psychoeducation on epilepsy. | Mo máa ń fún àwọn oní epilepsy ní ìmọ̀ ẹ̀kọ́ nípa àìlera wọn. |
| I assess suicide risk by asking directly. | Mo máa ń beèrè taara pé ṣé wọn ní ìfọkànbalẹ̀ pẹ̀lú ero ìpá-ẹni-ní-kú. |
| I document mental health cases using mhGAP forms. | Mo máa ń kọ gbogbo ọ̀rọ̀ àìlera ọpọlọ sílẹ̀ pẹ̀lú fọ́ọ̀mù mhGAP. |
| I counsel anxiety patients using mhGAP. | Mo máa ń gbìyànjú fún àwọn oní ìfọ̀kànbalẹ̀ pẹ̀lú àtòsọ́nà mhGAP. |
| I follow up on depressed patients. | Mo máa ń tẹ̀síwájú sí àwọn oní ìbànújẹ. |
| I collaborate on epilepsy management. | Mo máa ń ṣiṣẹ́ pọ̀ pẹlu àwọn mìíràn láti tọjú epilepsy. |
| I refer suicidal patients to support services. | Mo máa ń rán àwọn oní ero ìpá-ẹni-ní-kú lọ sí ilé ìrànlọ́wọ́. |

**4. Self-Efficacy Scale (Ìwọn Ìgbẹ́kẹ̀lé Ara ẹni)**

| **English Item** | **Yoruba Translation** |
| --- | --- |
| I am confident using the mhGAP flowchart for depression. | Mo ní ìgbẹ́kẹ̀lé pé mo le lò àtòsọ́nà mhGAP láti mọ ìbànújẹ. |
| I am confident managing epilepsy in PHC. | Mo ní ìgbẹ́kẹ̀lé pé mo le tọju epilepsy ní PHC. |
| I am confident assessing suicide risk. | Mo ní ìgbẹ́kẹ̀lé pé mo le fojú inú wo ewu ìpá-ẹni-ní-kú. |
| I can differentiate psychosis from other conditions. | Mo le yà psychosis kúrò nínú àwọn àìlera míì. |
| I can counsel anxiety-related complaints. | Mo ní ìfẹ́kúfẹ̀ pé mo le fìmọ̀ gbìyànjú fún àwọn tí wọn ní ìfọ̀kànbalẹ̀. |
| I can refer depression cases appropriately. | Mo le rán àwọn oní ìbànújẹ lọ síbi tí ó tọ́. |
| I can monitor epilepsy medication adherence. | Mo le tọ́jú pé àwọn oní epilepsy ń gba ogún wọn dáadáa. |
| I can respond to suicidal ideation appropriately. | Mo le fèsì tó yẹ sí ohun tí ènìyàn sọ pé ó fẹ́ pa ara rẹ̀. |
| I can manage psychosis using mhGAP flowcharts. | Mo le tọju psychosis pẹ̀lú àtòsọ́nà mhGAP. |
| I can provide psychoeducation for anxiety complaints. | Mo le kó ìmọ̀ tó péye fún àwọn tí wọn ní ìfọ̀kànbalẹ̀. |
